# Objective Evaluation of Out-of-Competition Volume of Action in Wheelchair Basketball Classification

**DOI:** 10.3390/sports13020048

**Published:** 2025-02-08

**Authors:** Yuki Shimoyama, Shintaro Kasai, Hiroaki Wagatsuma, Tatsuru Ibusuki, Takumi Tsukada, Kaori Tachibana

**Affiliations:** 1 Jichi Medical University Saitama Medical Center, Saitama 330-0834, Japan; y.shimoyama.gs@alumni.ipu.ac.jp; 2 Graduate School of Life Science and Systems Engineering, Kyushu Institute of Technology, Fukuoka 804-8550, Japan; kasai.shintaro660@mail.kyutech.jp (S.K.); waga@brain.kyutech.ac.jp (H.W.); 3 Akeno Central Hospital, Ooita 870-0161, Japan; tibusuki@akenohp.jp; 4 Institute of Sports Science and Environmental Physiology, Wakayama Medical University, Wakayama 640-8033, Japan; tennouhai14@gmail.com; 5 Graduate School of Health Sciences, Ibaraki Prefectural University of Health Sciences, Ibaraki 300-0331, Japan

**Keywords:** classification, wheelchair basketball, volume of action, physical impairment, sitting balance

## Abstract

In wheelchair basketball, classes are based on competition observations. Since 2021, out-of-competition testing has been implemented; however, research remains limited. This study aimed to determine whether the quantified volume of action (VOA) can be an indicator for classification and examined the influence of a competitive wheelchair on VOA evaluation. This cross-sectional study included 47 wheelchair basketball players (21 able-bodied, 26 with physical impairments: class 1, n = 8; class 2, n = 5; class 3, n = 4; class 4, n = 9). Tests were performed in a wheelchair (wheelchair condition) and on a trainer bed (bed condition). Participants held a ball and rotated their trunks in various planes. Movements were recorded using four cameras, and position coordinates were extracted using the three-dimensional DLT method. Classes and sitting conditions were compared across five groups: classes 1, 2, 3, 4, and able-bodied. Comparisons between classes revealed significant differences in all planes, including wheelchair and bed conditions (*p* < 0.05). The VOA expanded in the wheelchair condition compared to the bed condition across multiple classes and planes (*p* < 0.05). Measuring the VOA outside the competition while sitting on a bed may effectively classify players by eliminating equipment influence.

## 1. Introduction

The International Paralympic Committee (IPC) states that the purpose of classification in para-sports is to define who competes and to minimize the impact of eligible impairments in each event [1]. The current classification system for wheelchair basketball is based on the decision of a few experienced classifiers rather than empirical evidence, raising concerns about its validity. Therefore, researchers should explore the use of simple, readily available, objective, and less subjective instrumented measures [2].

The IPC classification code requires athletes to be allocated a sports class based on their ability to execute specific tasks and activities fundamental to the parasport or discipline [1]. The current International Wheelchair Basketball Federation (IWBF) classification involves submitting a medical certificate, physical function evaluation, movement confirmation on a platform, and observation and evaluation during practice and games in a wheelchair. Classes were divided into eight levels in descending order of physical impairment severity: 1.0, 1.5, 2.0, 2.5, 3.0, 3.5, 4.0, and 4.5 [3,4]. The classification system determines positions and roles on the court. A player in class 4.0 or 4.5 is suggested to have little difference from an able-bodied player regarding ability and role on the court [5].

The activity with the greatest impact on wheelchair basketball is volume of action (VOA), which involves controlled trunk movement in three planes of action [6]. VOA is the extent to which a player can move voluntarily in any direction and return to an upright seated position without holding the wheelchair for support or the upper extremities for aid. It includes all directions and describes the position of the ball as if the player were holding it with both hands. Players in each sports class have different VOAs, which are key elements of player evaluation. Class 1.0, which is the most severe physical impairment, has the smallest VOA. As the physical impairment lessens, the VOA increases, and since class 4.5 has the lightest physical impairment, it exhibits the largest VOA [3,4]. The classification manual defines VOA in the transverse, sagittal, and frontal planes, which refer to the vertical, forward, and sideways planes, respectively. A class 1.0 player has no VOA in either vertical, forward, or sideways planes. The class 1.0 player is primarily identified by passive trunk mobility in all activities, requiring frequent use of the upper extremities to maintain and adjust trunk position. A class 2.0 player has partial VOA in the vertical and forward planes but none in the sideways plane. A class 3.0 player has full VOA in the vertical and forward planes but none in the sideways plane. A class 4.0 player has complete VOA in the vertical and forward planes and on one side. A class 4.5 player has complete VOA in all planes, with no weakness in any direction. The class 4.5 player has all the attributes of a class 4.0 player but can control movement on both sides of the body, with no obvious weak or strong side [3,4].

Previous studies have reported the relationship between established classes and physiological indicators, such as heart rate and maximum oxygen uptake [7,8,9,10,11]. Marszałek et al. [7,8] reported differences in heart rate responses between low-pointers (classes 1.0–2.5) and high-pointers (classes 3.0–4.5). In addition, Molik et al. [9,10] conducted anaerobic power tests in eight groups and concluded that based on physiological criteria, the eight-level IWBF classification system should be reconsidered. However, De Lira et al. [11] reported that class correlated with aerobic and anaerobic performance, supporting the IWBF classification system. Other studies on the relationships between class and field-based performance tests have reported that throwing ability influences classification [12,13], while the current classification system reflects only a portion of performance ability [14]. Although research is being conducted into the relationship between current classes and physiological indicators and performance tests, no research has quantitatively evaluated VOA. This metric is a key element of player evaluation.

The IWBF Player Classification System classifies players with eligible physical impairments based on how their impairments affect fundamental wheelchair basketball activities. This classification is independent of skill level, athletic training, or equipment/technological differences [6]. However, wheelchair settings [15,16,17,18,19,20,21,22] and the use of straps can impact handling and trunk control [23,24,25,26,27]. Altmann et al. [23] reported that a growing issue in paralympic movement is the impact of equipment on performance, as athletes naturally seek to enhance their performance and minimize impairment effects using optimized equipment.

Despite these reports, during the classification procedure for wheelchair basketball, players are evaluated based on observations during practice and games in their wheelchairs. Tweedy et al. [2] reported that individualized adaptations, such as positioning and strapping, should not influence the class allocation of athletes. However, if athletes are permitted to use such adaptations, they can alter the nature of the activity. This may potentially reduce the impact of impairment and make comparison of results across participants problematic [2]. Mann et al. [27] stated that the classification system may require modification to minimize reliance on assessments of specific functional abilities. This is particularly important as advancements in equipment (e.g., new prosthetic limbs) could improve athlete functionality by better compensating for impairment and, consequently, change the nature of the impairment–performance relationship.

In wheelchair basketball, although multiple reports indicate that wheelchair settings and straps affect performance and classification, no reports regarding the impact of wheelchairs on VOA exist. Therefore, the purpose of this study was to clarify whether quantified VOA can be used as an indicator of classification. It also assessed the influence of competition wheelchairs on the quantitative evaluation of VOA. This study demonstrates that out-of-competition testing, in addition to competitive observation, may support the accurate classification of wheelchair basketball players. This study makes a significant contribution to the sports field by revealing that out-of-competition testing, in addition to competitive observation, may support the accurate classification of wheelchair basketball players.

## 2. Materials and Methods

### 2.1. Participants

Forty-seven wheelchair basketball players participated in this study, including 21 able-bodied players [AP] and 26 players with physical impairments [PP]. AP were those who did not meet the minimum physical impairment criteria for wheelchair basketball classification. Classification of PP was done by Classification Committee members certified by the Japan Wheelchair Basketball Federation. All players played wheelchair basketball for at least 1 year. Eight players had a classification of 1 or 1.5, five had a classification of 2 or 2.5, four had a classification of 3 or 3.5, and five had a classification of 4 or 4.5 (Table 1).

The study protocol was approved by the Human Ethics Review Committee of the Ibaraki Prefectural University of Health Sciences (Approval no.1063, e383) and conducted according to the principles of the Declaration of Helsinki. All participants provided written informed consent.

### 2.2. Procedure

The measurements were conducted indoors. Reflective markers were affixed to 23 locations on the body, task movements were photographed using four cameras (Lumix FX85D, Panasonic, Tokyo, Japan), and the images were synchronized using a synchronization lamp.

Measurements were conducted under two conditions: sitting at the top of the bed (bed condition) and sitting in a wheelchair (wheelchair condition). In the bed condition, participants sat deeply on a trainer bed and their thighs were secured with a cloth belt. A footrest was installed such that the hip and knee joints were flexed at 90° and the feet were secured to the footrest with a cloth belt. For the wheelchair condition, the participants used their regular competition wheelchairs with straps and other equipment secured during competitions. The wheelchair wheels were fixed using stoppers. The order of wheelchair condition and bed condition was randomly assigned.

**Figure 1:**
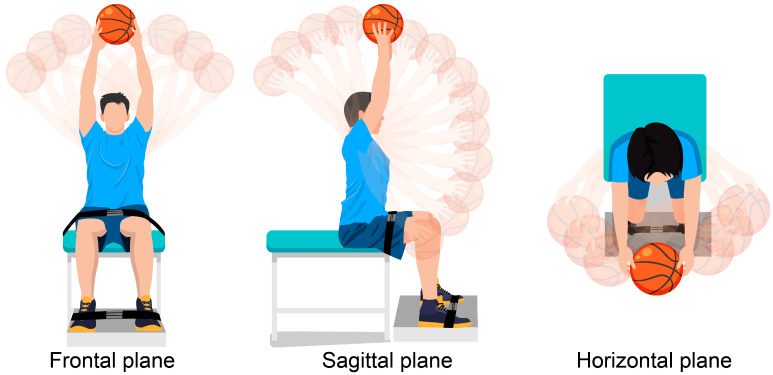
Test movements in the bed condition on each plane.

Participants were first briefed on the definition of VOA and instructed to hold a ball in both hands while moving their bodies as far as possible without gaining momentum. Verbal instructions were given to participants before the test. The researcher provided instructions for the movement simultaneously with their performance (Figure 1).

Regarding the speed of the movement, a metronome was not used because the same speed is perceived differently by each athlete depending on their disability. Tests were performed at a constant speed, which each athlete considered slow, and at which they could perform movements without momentum or compensatory movements.

The following four tests were conducted:

- trunk flexion: shoulder joint flexed at 180° to maximum trunk extension;
- trunk extension: shoulder joint flexed at 90° to maximum trunk extension;
- trunk lateral bending: maximum trunk lateral bending from left to right;
- trunk rotation: maximum trunk rotation from left to right.

Each test was performed twice consecutively, and data from the second test were used for analysis.

### 2.3. Data Acquisition and Analysis

We digitized the images captured with the four cameras and extracted the positional coordinates of the ball center point and body reference point using a three-dimensional video motion analysis system (Frame-DIAS VI, Q’sfix, Tokyo, Japan).

The center point of the ball was the midpoint between the left and right third metacarpal markers. The body reference point was the midpoint of the marker affixed to the intersection of the seat surface and the left and right back support pipes for the wheelchair condition and the midpoint of the reference marker affixed to the bed for the bed condition.

Based on the extracted coordinates, the trajectory of the ball was drawn using numerical analysis software (MATLAB2022a, version 2022a, MathWorks, Natick, MA, USA). The motion plane was derived using the least squares method. Subsequently, a curve approximation of the ball’s trajectory in the plane of motion was performed. The area of the sector formed by the approximate curve and intersection with the perpendicular line from the body reference point to the motion plane was calculated as VOA.

Considering the influence of sitting height and upper limb length in the sagittal and frontal planes, the area of a semicircle with the sum of the upper limb length and the distance between the seat surface and the acromion is the radius. In the horizontal plane, the area of a semicircle with the radius of the upper limb length was set to 100%, and the VOA was normalized.

### 2.4. Statistical Analyses

Participants were divided into five groups: class 1 (1.0 to 1.5), class 2 (2.0 to 2.5), class 3 (3.0 to 3.5), class 4 (4.0 to 4.5), and the AP, and statistical analysis were performed.

The assumption of normality was determined using the Shapiro–Wilk test for data within groups. For comparisons between classes, a one-way analysis of variance (ANOVA) was conducted separately for each group and each condition where data were normally distributed. The Kruskal–Wallis test was performed on items for which normality could not be assumed. Multiple comparisons were performed using Tukey’s test for items that showed significant differences in the one-way ANOVA and the Steel–Dwass test for items that showed significant differences in the Kruskal–Wallis test. The effect size, *r*, was calculated using the following formula based on the *Z* value calculated with the *p*-value of the Wilcoxon signed-rank test:

$$r=\frac{Z}{\sqrt{n}}$$

Regarding the frontal plane, the classification manual states that only class 4.5 has complete trunk movements on both sides, including bilateral lateral planes. Therefore, we conducted an additional statistical analysis in the six groups, dividing class 4 into class 4.0 and class 4.5.

For comparisons between sitting conditions, paired *t*-tests were performed for classes that were found to be normal. The Wilcoxon signed-rank test was performed for classes where normality could not be assumed. The effect size, *d*, was calculated using the following formula:

$$d=\frac{Mean\;of\;wheelchair\;condition\;-\;SD\;of\;bed\;condition}{\sqrt{\frac{{SD\;of\;wheelchair\;condition}^{2}-{SD\;of\;bed\;condition}^{2}}{2}}}$$

The level of significance was set at *p* < 0.05.

The effect size was evaluated using *d* and *r*, with d values of 0.2, 0.5, and 0.8 reflecting small, medium, and large effects, and *r* values of 0.1, 0.3, and 0.5 reflecting small, medium, and large effects, respectively [28].

Data analysis was performed using RStudio, Version 2023.06.2+561 (Certified B Corporation^®^, Boston, MA, USA).

## 3. Results

The mean and standard deviation of VOA for each class in the horizontal, sagittal, and frontal planes and in the sitting position are shown (Table 2).

In the bed condition, VOA gradually expanded from class 1 to AP in the horizontal and frontal planes. In the frontal plane, VOA gradually expanded from class 1 to class 4, and the results for AP were marginally smaller than those for class 4.

In the wheelchair condition, VOA gradually increased in the horizontal plane from class 1 to the AP, similar to the edge-sitting condition. In the sagittal plane, the difference between class 1 and class 2 was the largest. VOA gradually expanded from class 2 to class 4, and the AP was marginally smaller than class 4. In the frontal plane, similar to the sagittal plane, the largest difference was observed between class 1 and class 2. The difference remained consistent from class 2 to the AP.

### 3.1. Comparisons Between Classes

Based on the results of the Shapiro–Wilk test, a one-way ANOVA was performed on the sagittal plane in the wheelchair condition. The Kruskal–Wallis test was performed on the horizontal and frontal planes for the wheelchair condition. It was also performed on the horizontal, sagittal, and frontal planes for the bed condition.

Significant differences were observed in all planes for both wheelchair and bed conditions (bed condition: horizontal plane, *p* < 0.001, sagittal plane, *p* < 0.001, frontal plane, *p* < 0.001, wheelchair condition: horizontal plane, *p* < 0.001, sagittal plane, *p* < 0.001, frontal plane, *p* < 0.001).

**Figure 2:**
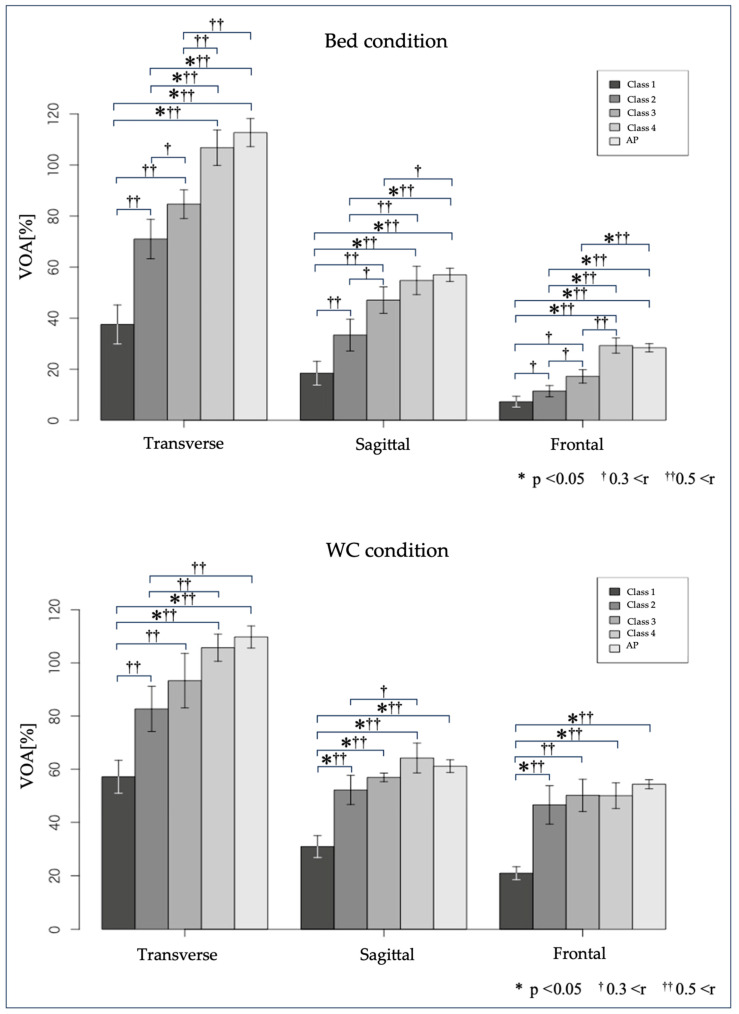
Comparisons between classes. VOA, volume of action.

Tukey’s and Steel–Dwass tests were performed for multiple comparisons. The effect size, *r*, was calculated, and the results are shown in Table 3 and Figure 2.

Regarding the frontal plane, the classification manual states that only class 4.5 has complete trunk movements both sides, including bilateral lateral planes. Therefore, we conducted an additional statistical analysis in the six groups, dividing class 4 into class 4.0 and class 4.5. Significant differences were observed in all planes for both wheelchair and bed conditions (bed condition: *p* < 0.001; wheelchair condition: *p* < 0.001).

The Steel–Dwass test was performed for multiple comparisons. The effect size, *r*, was calculated, and the results are shown in Table 3.

### 3.2. Comparisons Between Sitting Conditions

**Figure 3:**
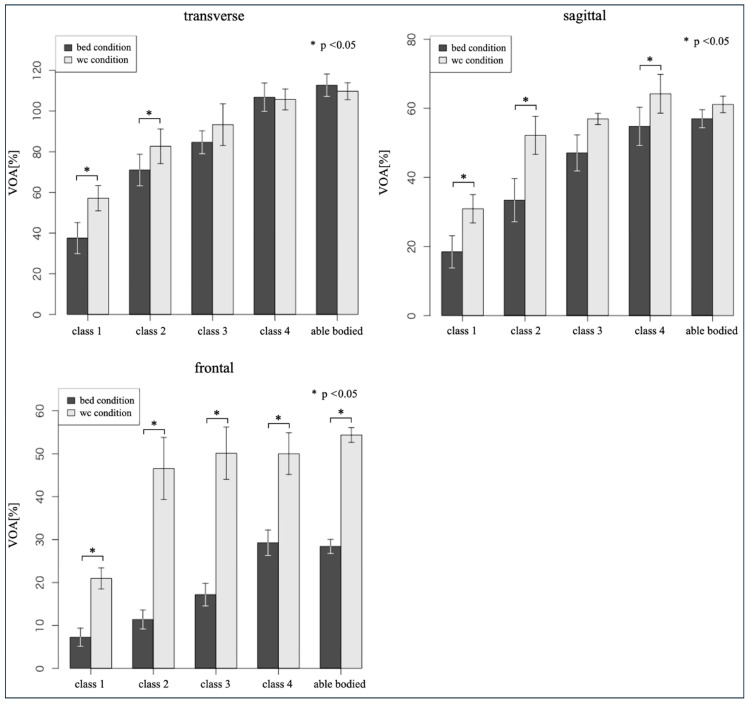
Comparisons between sitting conditions.

Based on the results of the Shapiro–Wilk test, the Wilcoxon signed-rank test was performed on the frontal plane in class 4 and on all planes in the AP. For other variables, paired *t*-tests were performed and effect sizes (d) were calculated. The results are presented in Table 3 and Figure 3.

A statistically significant difference (*p* < 0.05) was observed in the horizontal plane between class 1 and class 2 and in the sagittal plane between class 1, class 2, and class 4. In the frontal plane, statistically significant differences (*p* < 0.05) were found across all aspects, including the AP. Although no statistically significant difference was observed in class 3 in the sagittal plane, a large effect size was observed.

When measuring VOA under wheelchair conditions, larger VOAs were observed in class 1 and class 2 on the horizontal plane than under bed conditions. In the sagittal plane, larger VOAs were calculated in classes 1–4, excluding AP, than in the bed condition. In the frontal plane, larger VOAs were observed in all groups, including AP, when measuring VOA in the wheelchair than in the bed condition.

## 4. Discussion

This study aimed to provide valuable insights into the classification process in wheelchair basketball, particularly regarding the potential use of VOA as a quantifiable indicator. The significant differences observed in VOA across different classes, both in wheelchair and bed conditions, highlight this metric’s ability to distinguish between varying levels of physical impairment. Moreover, the expansion of VOA in the wheelchair condition compared to the bed condition suggests that competitive equipment can influence the assessment, underscoring the importance of controlling for such variables when classifying players.

### 4.1. Comparisons Between Classes

In the interclass comparison of normalized VOA, no statistically significant differences were found in the wheelchair condition. However, many groups with moderate or large effect sizes were observed in the bed condition. Although no statistically significant difference was found, items with moderate or large effect sizes were commonly related to class 3, which had fewer participants (n = 4). The effect size was not affected by the number of participants [28]. Therefore, although no statistically significant differences were observed for items with moderate or large effect sizes, they may become apparent with a larger sample size.

The VOA measurements under wheelchair conditions revealed class 1 characteristics. Under bed conditions, it was possible to recognize the characteristics of class 1, class 2, and class 3 VOA in the horizontal and frontal planes. In the sagittal plane, the characteristics of class 1 and class 2 VOA were recognized.

The definitions of VOA for each class in the classification manual are provided in Table A1.

In the wheelchair condition, only class 1 differed from the other classes in all planes. As there are no differences in VOA characteristics between class 2, class 3, class 4, and APs, evaluating the VOA of each plane by observing a wheelchair competition may be challenging.

In the bed condition, we observed the differences between class 1, class 2, and class 3 compared to the other classes on the horizontal plane. The manual states that classes 3 and above have complete trunk movement in the horizontal plane; however, differences between class 3 and class 4 and above can be confirmed through out-of-competition testing in the edge-sitting position. For the VOA in the sagittal plane, classes 3 and above have complete trunk movement. In the bed condition, the differences between class 1 and class 2 from other classes were recognized. Differences between class 2, class 3, and other classes, which are difficult to confirm during competitions using a wheelchair, can be confirmed through out-of-competition testing in a sitting position.

In the statistical analysis and effect size of six groups, dividing class 4.0 and class 4.5 in the frontal plane, we recognized differences between class 1, class 2, and class 3 compared to other classes when comparing the bed condition. “Small” effect sizes should not be so significant that they are obscured by measurement and experimental biases, and not so large that they are easily perceptible to the naked eye [28]. While comparing frontal plane VOA between class 4.0, class 4.5, and the AP, a small effect size was confirmed between class 4.0 and class 4.5 and between class 4.0 and the AP in the bed condition. However, no significant effect size was observed between class 4.5 and the AP (class 4.0–class 4.5: r = 0.200; class 4.0–AP: r = 0.279; class 4.5–AP: r = 0.071). In the wheelchair condition, a medium effect size was observed between class 4.0 and class 4.5. A small effect size was observed between class 4.0 and the AP. Even a small effect size was not observed between class 4.5 and the AP (class 4.0–class 4.5: r = 0.374; class 4.0–AP: r = 0.267; class 4.5–AP: r = 0.071).

In the frontal plane, the absence of a significant effect size for class 4.5 compared to AP in both the bed and wheelchair conditions aligns with the manual’s description that “Class 4.5 has complete trunk movements to both sides”. Additionally, the small and medium effect sizes observed between class 4.0 and class 4.5 indicate that these classes differ. These results suggest that VOA measurements taken while sitting on a bed can be used to classify wheelchair basketball. Measuring VOA using a wheelchair makes it possible to distinguish between class 1 and other classes; however, it is difficult to distinguish between class 2, class 3, class 4, and the AP.

### 4.2. Comparisons Between Sitting Condition

We compared the sitting conditions of VOA normalized using the participants’ upper limb lengths and the distance between the seat surface and the acromion.

In the horizontal plane, a larger VOA was measured in the wheelchair condition than in the bed condition for all items except class 4 and the AP. Regarding the two items where a larger VOA was measured in the bed condition, no statistically significant difference was observed, and even the effect size (small) was not calculated. Therefore, the difference in sitting conditions had little effect on the VOA (class 4: *p* = 0.0780, d = −0.06; AP: *p* = 0.0179, d = −0.16).

In the transverse plane, a statistically significant difference and a large effect size were observed between class 1 and class 2 (class 1: *p* = 0.0009, d = 1.00; class 2: *p* = 0.044, d = 9.44). Regarding VOA in the horizontal plane, classes 3 and above had complete trunk movement. Competition wheelchairs may have also amplified the incomplete VOA of class 1 and class 2. Curtis et al. [26] compared trunk movement and functional reach in wheelchairs using no belt, chest belt, or thigh belt. They hypothesized that using either a chest or thigh belt helps stabilize the trunk and pelvis, respectively, while increasing functional reach. Their results were consistent with their assumptions. We believe that the increase in VOA in the transverse plane of this study was for a similar reason.

In the sagittal plane, a statistically significant difference was observed among class 1, class 2, and class 4, a large effect size was observed for class 1 and class 2, and a medium effect size was observed for class 4 (class 1: *p* = 0.0002, d = 1.00; class 2: *p* = 0.009, d = 1.43; class 4: *p* = 0.005, d = 0.56). No statistically significant difference was observed in class 3. This class had a large effect size and was the group with the smallest number of participants (*p* = 0.094, d = 1.27). Thus, a statistically significant difference may be observed as the number of participants increases. VOA in the sagittal plane shows complete trunk movement in classes 3 and above, similar to the transverse plane. However, we observed wheelchair-expanded VOA in classes 1– 4. In the AP, no statistically significant difference was observed; however, the effect size was small (*p* = 0.065, d = 0.48). This result suggests that even in healthy AP, competition wheelchairs may have a minor effect on VOA in the sagittal plane that is not easily visible. The VOA in the sagittal plane appears more susceptible to the influence of competition wheelchairs than in the horizontal plane.

In the frontal plane, statistically significant differences and large effect sizes were observed in all classes, including the AP (class 1: *p* < 0.0001, d = 2.12; class 2: *p* = 0.004, d = 2.95; class 3: *p* = 0.016, d = 6.24; class 4: *p* = 0.004, d = 1.72; AP: *p* < 0.001, d = 3.55). Wheelchairs affect the frontal plane the most. We speculate that this was due to the fixation and support provided by the side guards and straps.

Marszalek et al. [25] measured the trunk momentum in terms of angle and compared the results between classes with and without straps, showing that the use of a strap increased trunk movement in each plane, which is consistent with the results of this study. They also reported that trunk flexion and rotation discriminated between the most effective players. We speculated that this was due to trunk momentum, which was measured with both hands crossed in front of the chest. In wheelchair basketball games, such as when competing for a rebound, the range of motion in the elbow extension position is important. Hence, we believe that the range of motion in the frontal plane, including trunk momentum, is also important for classification.

Wheelchairs expanded the VOA across most classes and planes. Based on the study results, wheelchairs have the potential to increase VOA. Thus, measuring VOA while sitting at the upper end of the bed is effective for classifying players while minimizing the influence of wheelchairs.

### 4.3. Limitations and Future Research

The first limitation of this study was the small number of participants. When the results were compared by dividing them into five groups: class 1, class 2, class 3, class 4, and the AP, the number of participants per group was small. In particular, class 3 had the smallest number of participants (n = 4), and it was unclear whether participant data matched the generalized data. Although no statistically significant differences were observed, multiple differences were noted among the groups in which a medium or large effect size was calculated. We believe that as the number increases, a statistically significant difference may be observed. In this study, we compared five groups; however, wheelchair basketball classes were divided into eight levels in 0.5 increments. Increasing the number of participants and investigating whether more detailed class characteristics can be recognized is necessary. Furthermore, the small number of female participants and the variation in gender ratio hindered further characterization of the classes.

In addition, participants could intentionally make task movements smaller. Although we quantified VOA and recognized the characteristics of different classes, elucidating the characteristics of each class and identifying indicators of cheating is necessary for detecting intentional fraud in classification.

Finally, this study collected and analyzed data focusing on VOA, which is reported to have the greatest influence on wheelchair basketball performance [6]. Consequently, the currently determined class reflected the measured and quantified VOA in the edge-sitting position. Wheelchair basketball performance is affected by various factors, including VOA, acceleration, propulsive action, balance preservation, and braking [6,29,30,31]. Therefore, although VOA cannot determine a class alone, objective measurement of VOA will aid in evidence-based classification. Recently, markerless motion capture has also been developed, making it possible to analyze the performance of extreme poses, such as lowering the head [32]. This study may help guide objective measurements in the future.

## 5. Conclusions

It has been suggested that measuring VOA on a bed outside of competition may help classify players by eliminating the influence of equipment. In particular, measuring VOA in a standardized environment, rather than in a competition wheelchair tailored to each athlete, makes class differences more apparent. In addition to observing competitions in a wheelchair, out-of-competition testing is thought to be helpful in accurately classifying players in wheelchair basketball.

## Figures and Tables

**Table 1:** Participants characteristics (n = 47).

Class	n	Wheelchair Basketball Experience (y)	Gender (M:F)	Age (y)
1.0	5	13.2 ± 7.3	4:1	32.8 ± 9.5
1.5	3	29.0 ± 12.1	2:1	50.3 ± 18.2
2.0	2	4.5 ± 4.9	1:1	40.0 ± 7.1
2.5	3	14.0 ± 3.5	2:1	37.7 ± 5.1
3.0	2	14.5 ± 2.1	1:1	29.5 ± 2.1
3.5	2	17.0 ± 9.9	2:0	38.0 ± 17.0
4.0	3	10.7 ± 8.3	1:2	25.0 ± 4.4
4.5	6	12.4 ± 6.8	2:4	37.0 ± 8.3
AP	21	3.9 ± 2.8	11:10	22.7 ± 2.6
Total	47	9.4 ± 8.5	16:21	30.1 ± 10.6

**Table 2:** Mean and standard deviation of VOA.

	Bed Condition			WC Condition		
	Transverse	Sagittal	Frontal	Transverse	Sagittal	Frontal
Class 1	37.55	18.45	7.25	57.15	30.92	20.96
	(21.58)	(13.21)	(5.98)	(17.53)	(11.56)	(6.92)
Class 2	71.02	33.39	11.39	82.68	52.17	46.56
	(17.30)	(13.96)	(4.95)	(19.02)	(12.29)	(16.12)
Class 3	84.68	47.09	17.18	93.33	56.91	50.11
	(11.25)	(10.42)	(5.28)	(20.51)	(3.23)	(12.13)
Class 4	106.81	54.78	29.28	105.72	64.21	50.01
	(20.84)	(16.60)	(8.92)	(15.39)	(16.85)	(14.48)
AP	112.73	56.99	28.42	109.77	61.12	54.32
	(25.23)	(11.98)	(7.51)	(19.07)	(10.90)	(7.86)

Upper = mean, Lower = SD. AP, able-bodied players; VOA, volume of action.

**Table 3:** Results of multiple comparisons between classes and effect size.

		Bed Condition					WC Condition				
		Class 1	Class 2	Class 3	Class 4	Class 4.5	Class 1	Class 2	Class 3	Class 4	Class 4.5
Transverse	Class 2	0.093	-	-	-	-	0.243	-	-	-	-
		(0.706) ^††^					(0.556) ^††^				
	Class 3	0.051	0.737	-	-	-	0.121	0.864	-	-	-
		(0.830) ^††^	(0.356) ^†^				(0.694) ^††^	(0.273)			
	Class 4	0.005 *	0.049 *	0.817	-	-	0.007 *	0.235	0.817	-	-
		(0.955) ^††^	(0.769) ^††^	(0.543) ^††^			(0.914) ^††^	(0.544) ^††^	(0.270)		
	AP	0.000 *	0.013 *	0.716	0.997	-	0.000 *	0.090	0.716	0.997	-
		(0.911) ^††^	(0.699) ^††^	(0.527) ^††^	(0.155)		(0.936) ^††^	(0.506) ^††^	(0.239)	(0.057)	
Sagittal	Class 2	0.316	-	-	-	-	0.029 *	-	-	-	-
		(0.511) ^††^					(0.652) ^††^				
	Class 3	0.080	0.737	-	-	-	0.010 *	0.977	-	-	-
		(0.765) ^††^	(0.356) ^†^				(0.830) ^††^	(0.196)			
	Class 4	0.009	0.300	0.066	-	-	0.000 *	0.402	0.855	-	-
		(0.871) ^††^	(0.503) ^††^	(0.186)			(0.914) ^††^	(0.314) ^†^	(0.061)		
	AP	0.000 *	0.031 *	0.048 *	1.000	-	0.000 *	0.582	0.968	0.968	-
		(0.911) ^††^	(0.611) ^††^	(0.333) ^†^	(0.024)		(0.911) ^††^	(0.251)	(0.122)	(0.032)	
Frontal	Class 2	0.586	-	-	-	-	0.043 *	-	-	-	-
		(0.380) ^†^					(0.820) ^††^				
	Class 3	0.177	0.582	-	-	-	0.051	0.999	-	-	-
		(0.380) ^†^	(0.436) ^†^				(0.830) ^††^	(0.040)			
	Class 4	0.005 *	0.023 *	0.066	-	-	0.007 *	0.990	1.000	-	-
		(0.955) ^††^	(0.880) ^††^	(0.768) ^††^			(0.914) ^††^	(0.103)	(0.021)		
	AP	0.000 *	0.009 *	0.048 *	0.993	-	0.000 *	0.905	0.985	0.847	-
		(0.911) ^††^	(0.754) ^††^	(0.597) ^††^	(0.073)		(0.936) ^††^	(0.162)	(0.093)	(0.179)	
Frontal	Class 2	0.687	-	-	-	-	0.060	-	-	-	-
in 6 groups		(0.380) ^†^					(0.820) ^††^				
	Class 3	0.234	0.684	-	-	-	0.072	1.000	-	-	-
		(0.633) ^††^	(0.436)				(0.830) ^††^	(0.040)			
	Class 4.0	0.140	0.221	0.276	-	-	0.217	0.998	0.897	-	-
		(0.756) ^††^	(0.743) ^††^	(0.597) ^††^			(0.679) ^††^	(0.096)	(0.318) ^†^		
	Class 4.5	0.024 *	0.068	0.176	1.000	-	0.024 *	0.943	0.998	0.790	-
		(0.910) ^††^	(0.860) ^††^	(0.820) ^††^	(0.200)		(0.910) ^††^	(0.239)	(0.096)	(0.374) ^†^	
	AP	0.001 *	0.013 *	0.067	0.999	1.000	0.001 *	0.952	0.995	0.755	0.999
		(0.911) ^††^	(0.754) ^††^	(0.597) ^††^	(0.279)	(0.071)	(0.936) ^††^	(0.162)	(0.093)	(0.261)	(0.071)

Upper = *p* value, Lower = effect size r; * *p* <0.05; ^†^ 0.3 < r; ^††^ 0.5 < r. AP, able-bodied players; VOA, volume of action.

## Data Availability

The data presented in this study are available on request from the corresponding author due to privacy protection. Please contact the corresponding author (K.T.) for additional steps to acquire the data.

## Appendix A

**Table A1:** The classification manual defines VOA in the transverse, sagittal, and frontal planes as being expressed as a vertical plane, a forward plane, and a sideways plane, respectively.

Class	Characteristics of VOA
class 1	Has no volume of action in either the vertical, forward, or sideways planes.
	Primarily identified by passive mobility of the trunk in all activities, requiring frequent use of the upper extremities to maintain and adjust trunk position.
class 2	Has a partial volume of action in the vertical and forward planes but no volume of action in the sideway plane.
class 3	Has full volume of action in the vertical and forward planes, but no volume of action in the sideway plane.
class 4.0	Has complete volume of action in the vertical and forward planes and complete volume of action to only one side.
class 4.5	Has complete volume of action in all planes, with no weakness in any direction.
	Has all the attributes of the class 4.0 player, but it can control movement to both sides of the body. Has no obvious weak or strong side.

vertical plane = transverse plane, forward plane = sagittal plane, sideways plane = frontal plane.
